# Antenatal Presentation of MRPS22‐Related Mitochondrial Disease Confirmed With Rapid Proteomics

**DOI:** 10.1002/jmd2.70092

**Published:** 2026-04-24

**Authors:** Liana N. Semcesen, Megan Ball, Daniella H. Hock, Juliet Kaye, Aimee Woods, Lucas De Jong, MitoMDT Diagnostic Network for Genomics and Omics, David R. Thorburn, Aleksandra Filipovska, Michael T. Ryan, David A. Stroud, Diana Stojanovski, David Coman, Sean Murray, Ryan L. Davis, John Christodoulou, Suzanne C. E. H. Sallevelt, Roula Ghaoui, Cas Simons, Stefan J. Siira, Shanti Balasubramaniam, Alison G. Compton, Daniel G. MacArthur, Nicole J. Lake, Drago Bratkovic, Joy Lee, Maina Kava, Amanda Samarasinghe, Yoni Elbaum, Catherine Atthow, Pauline McGrath, Tegan Stait, Rocio Rius, Liana N. Semcesen, Megan Ball, Daniella Hock, Luke E. Formosa, Ellenore M. Martin, Madeleine Harris, John Christodoulou, David R. Thorburn, Alison G. Compton, David A. Stroud, Jan Liebelt

**Affiliations:** ^1^ Department of Biochemistry and Pharmacology Bio21 Molecular Science and Biotechnology Institute, University of Melbourne Parkville Victoria Australia; ^2^ Murdoch Children's Research Institute Melbourne Victoria Australia; ^3^ Department of Paediatrics University of Melbourne Melbourne Victoria Australia; ^4^ Royal Children's Hospital Melbourne Victoria Australia; ^5^ Victorian Clinical Genetics Services Murdoch Children's Research Institute Melbourne Victoria Australia; ^6^ Medical Imaging Women's and Children's Hospital Adelaide South Australia Australia; ^7^ Maternal Fetal Medicine Department Women's and Children's Hospital Adelaide South Australia Australia; ^8^ Genetics and Molecular Pathology SA Pathology Adelaide South Australia Australia; ^9^ SA Clinical Genetics Service Women's and Children's Hospital Adelaide South Australia Australia; ^10^ Repromed (Adelaide Fertility Centre) Adelaide South Australia Australia

**Keywords:** corpus callosum, genomic autopsy, hydrops fetalis, mitochondrial disease, mitoribosome, MRPS22, proteomics

## Abstract

*MRPS22*‐related mitochondrial disease (MIM#611719) is a rare autosomal recessive disorder caused by defects in the mitochondrial ribosomal protein S22, a component of the small mitoribosomal subunit essential for mitochondrial translation. Of the few reported cases, most present antenatally with a severe phenotype, conveying a poor prognosis. We describe a fetus with severe antenatal‐onset *MRPS22*‐related mitochondrial disease and the use of multi‐omics in the molecular diagnosis. A primigravida underwent termination of pregnancy following identification of multiple congenital anomalies (hydrops fetalis, microcephaly, corpus callosal agenesis, periventricular cysts and cardiac hypertrophy) on ultrasound at 20 + 2 weeks' gestation, confirmed on fetal magnetic resonance imaging. Trio genome sequencing revealed compound heterozygous variants in *MRPS22* (NM_020191.4: c.509G>A; p.(Arg170His) and c.565C>G; p.(Arg189Gly)). Rapid proteomic analysis demonstrated destabilisation of the small mitoribosomal subunit and combined reduction of OXPHOS complexes, supporting the pathogenicity of the variants. This case consolidates the antenatal phenotype of severe MRPS22‐related disease and highlights the importance of considering mitochondrial disease in the differential diagnosis of congenital anomalies, especially hydrops fetalis and corpus callosum anomalies. This study provides evidence for the utility of multi‐omic approaches (trio genome sequencing, proteomics) in confirming variant pathogenicity following pregnancy loss, enabling accurate diagnosis, and informing reproductive counselling for affected families.

## Key Points

- *MRPS22*‐related mitochondrial disease should be considered in the differential diagnosis of fetal hydrops and multiple congenital anomalies, particularly in the presence of corpus callosum agenesis.
- Rapid proteomic analysis confirmed the pathogenicity of *MRPS22* variants identified by genomic autopsy, demonstrating the utility of multi‐omic diagnostics following pregnancy loss.

## 1 Introduction

Mitochondrial diseases are a group of disorders that impact mitochondrial energy generation, either by directly affecting oxidative phosphorylation (OXPHOS) or indirectly affecting other mitochondrial pathways or functions [1]. The OXPHOS system is comprised of five multimeric protein complexes (CI‐V), whose protein subunits are under dual genomic control, being encoded by the nuclear and mitochondrial genomes (mtDNA). Mitochondrial ribosomes (mitoribosomes) are essential for the translation of the 13 mtDNA‐encoded OXPHOS proteins. The human mitoribosome consists of a 28S small mitoribosomal subunit (mtSSU), containing 30 mitoribosomal proteins (MRPs) and the 12S mt‐rRNA, and a 39S large mitoribosomal subunit (mtLSU), containing 52 MRPs, the 16S mt‐rRNA, and the mitochondrial tRNA^val^ [2, 3]. MRPs are encoded by the nuclear genome, translated in the cytosol, and imported into the mitochondria for mitoribosome assembly. Many MRPs have been associated with mitochondrial disease and encompass a broad clinical spectrum of presentations [4].

*MRPS22* encodes the mitochondrial ribosomal protein small 22 (MRPS22), a mtSSU subunit incorporated early in its assembly [3]. To date, biallelic variants in *MRPS22* have been described in at least 11 cases from six families with severe, early onset mitochondrial disease (Combined Oxidative Phosphorylation Deficiency 5, MIM#611719), frequently presenting in utero [5, 6, 7, 8, 9, 10]. *MRPS22* has also been associated with a milder phenotype of Premature Ovarian Insufficiency with or without peripheral neuropathy (Ovarian Dysgenesis 7, OMIM#618117) [11, 12]. Here we present a case of severe antenatal‐onset disease characterised by hydrops, brain anomalies, microcephaly and cardiac hypertrophy caused by biallelic *MRPS22* variants, supported by proteomic evidence of combined OXPHOS deficiency consistent with disrupted mtSSU assembly. We also review the literature to delineate the clinical spectrum of early‐onset MRPS22‐related mitochondrial disease.

## 2 Results

### 2.1 Phenotype

**FIGURE 1:**
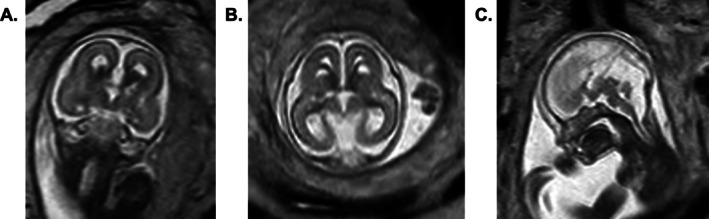
Antenatal magnetic resonance imaging (Philips GE 1.5) at 21 + 2 weeks gestation. (A) Coronal T2 demonstrating callosal agenesis, cystic spaces within the basal ganglia. (B) Axial T2 demonstrating cystic spaces within the caudothalamic eminences, thalami, and periventricular white matter. (C) Sagittal T2 demonstrating callosal agenesis and inferior vermian hypoplasia.

A 35‐year‐old primigravida was referred to the Adelaide Women's and Children's Hospital Maternal Fetal Medicine Unit in the context of significant congenital anomalies. The parents were a healthy non‐consanguineous couple of European ancestry, with no significant family history. Morphological ultrasound at 20 + 2 weeks of gestation identified the fetus was microcephalic (HC 157.2 mm, 7%, BPD 41.6 mm, < 1%) and small for gestational age. There was prominence of the lateral ventricles, each measuring 9 mm, with colpocephaly. There was a midline cystic structure, measuring 11 × 4 mm. The corpus callosum and cavum septi pellucidi were not visualised. The fetus was hydropic with cystic hygroma, bilateral pleural effusions, pericardial effusion and ascites. Fetal middle cerebral artery (MCA) dopplers showed an elevated MCA peak systolic velocity (38.8 cm/s, MOM 1.50). The ventricular myocardium appeared thickened with an increased cardiothoracic ratio (97th centile), suggestive of cardiac hypertrophy. Fetal magnetic resonance imaging at 21 + 2 weeks of gestation confirmed significant intracranial anomalies (complete agenesis of the corpus callosum, small cerebellum and vermis, hypoplastic inferior vermis, prominent cisterna magna, cystic changes in the basal ganglia, thalami and cerebral parenchyma, Figure 1). Prenatal microarray on amniotic fluid showed a female profile, with no clinically significant abnormality. Maternal viral screen and cytomegalovirus PCR on amniotic fluid was negative. Following these findings, the couple made the difficult decision to not continue the pregnancy, and termination of pregnancy occurred at 21 + 5 weeks of gestation.

### 2.2 Autopsy Findings

Autopsy confirmed the antenatal findings of hydrops (subcutaneous oedema, pleural and pericardial effusions, ascites), intracranial anomalies (microcephaly, agenesis of the corpus callosum, periventricular cystic changes, multiple targetoid haemorrhagic lesions over both cerebral hemispheres and extensive intracranial haemorrhage) and thickened myocardium (normal weight for gestation) with mildly disordered myofibres on histology. Reduced organ weight (pulmonary hypoplasia, small thymus, and liver < 5th centile) and mild renal pelvicalyceal dilatation were noted. Testing for toxoplasmosis and cytomegalovirus did not identify any evidence of infection. Post‐mortem dried blood spot tandem mass spectrometry exhibited characteristic post‐mortem amino acid and acyl‐carnitine profiles.

### 2.3 Genotype

Trio genome sequencing was performed post‐mortem, which identified the fetus was compound heterozygous for a paternally inherited likely pathogenic variant, NM_020191.4:c.509G>A; p.(Arg170His) and a maternally inherited variant of uncertain significance (VUS), NM_020191.4:c.565C>G; p.(Arg189Gly) in the nuclear‐encoded mitochondrial disease gene, *MRPS22*. The c.509G>A variant is present at low frequency in population databases (gnomAD v4: 234 heterozygotes and 0 homozygotes). The p.Arg170 residue is highly conserved, and the variant is predicted to be damaging by *in silico* analyses (REVEL = 0.91). It has been reported in homozygous state in three affected siblings [5] and in compound heterozygous state in other affected individuals (ClinVar Accession: VCV000004753.10) [6]. Respiratory chain enzyme (Complexes I, III, IV and V) activity was reported to be reduced in muscle in an affected individual (8%–30% of the control mean). Significant reduction of 12S rRNA and other small ribosomal subunits was reported in patient fibroblasts [5, 13]. When patient cells were transfected with wildtype *MRPS22* cDNA, the 12S rRNA content was increased and respiratory chain enzyme activity was normalised [5]. The c.565C>G variant is ultra rare in population databases (gnomAD v4: 2 heterozygotes and 0 homozygotes) and has not previously been reported in affected individuals. The p.Arg189 residue is highly conserved, and the variant is predicted to be damaging by *in silico* analyses (REVEL = 0.87).

### 2.4 Proteomics

**FIGURE 2:**
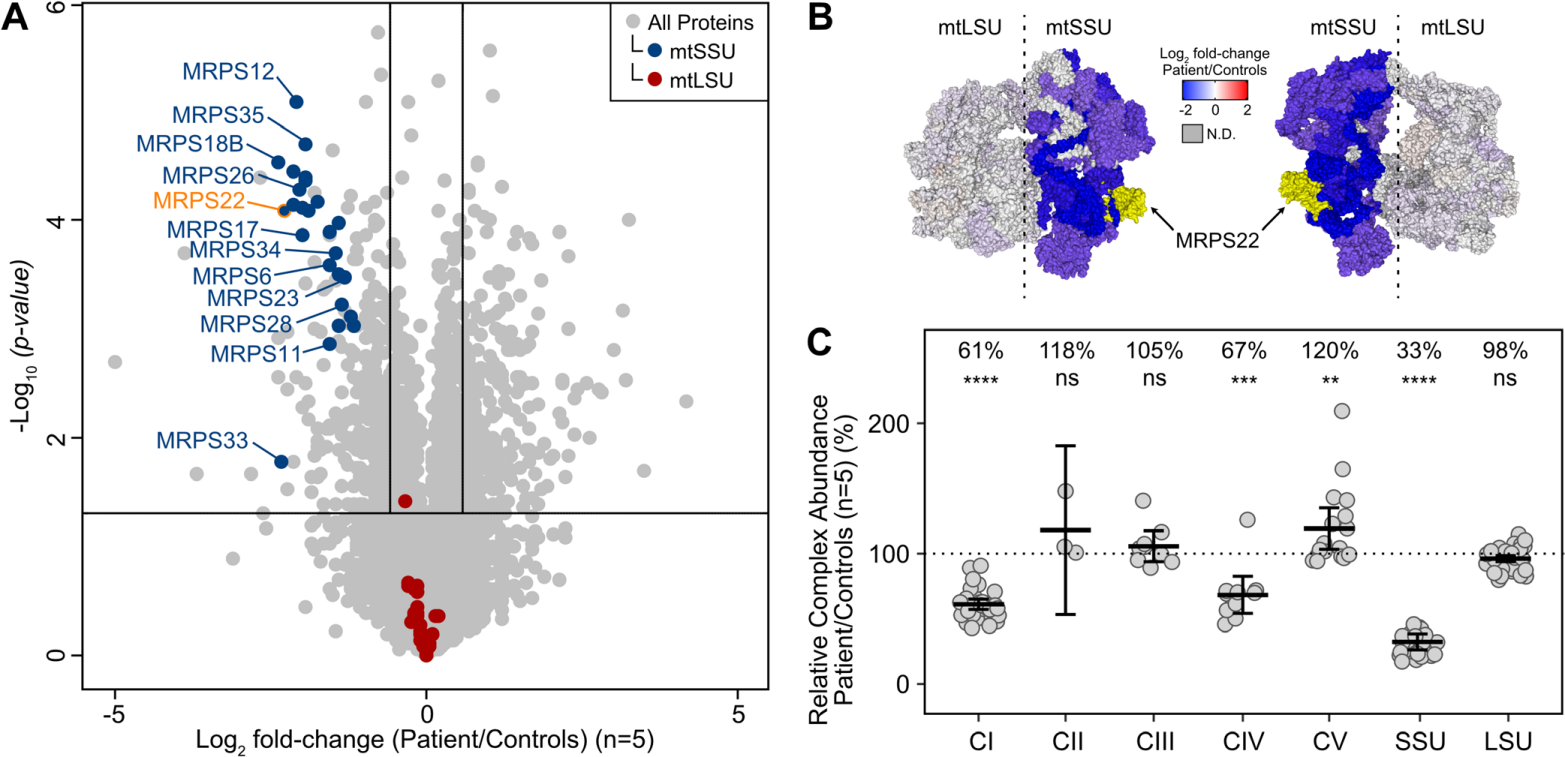
(A) Volcano plot showing abundances of all cellular proteins detected in proband fibroblasts compared to controls (*n* = 5) by quantitative proteomics analysis, demonstrating reduced abundances of mitoribosome small subunit (mtSSU) proteins in the proband. Vertical lines represent ±1.5 fold‐change (log_2_ = ±0.585) and horizontal line represents *p‐*value = 0.05 (−log_10_ = 1.301). Blue = mitoribosome small subunit (mtSSU). Red = mitoribosome large subunit (mtLSU). (B) Topographical heatmap of mitoribosome proteins showing log_2_ fold‐changes of each subunit in proband fibroblasts relative to controls, mapped onto the cryo‐EM structure of the human mitoribosome. MRPS22 indicated with an arrow. N.D = not detected. Yellow = MRPS22. PDB = 3J9M. (C) Relative Complex Abundance (RCA) of OXPHOS Complexes I–V (CI‐V) and mitoribosome small (SSU) and large (LSU) subunits within proband fibroblasts, based on the mean ratio of the proteins within the complex relative to controls. Significance was determined by a paired *t‐*test. ** = *p* < 0.01, *** = *p* < 0.001, **** = *p* < 0.0001, ns = not significant.

Untargeted quantitative proteomics was performed on whole cell fibroblast lysates from the proband and five paediatric control individuals to elucidate the effects of the *MRPS22* variants on global proteome levels. The abundance of all detected proteins comprising the mtSSU was significantly decreased in proband fibroblasts when compared to controls, including MRPS22 protein. In comparison, the abundance of all detected proteins of the mtLSU was unchanged (Figure 2A). Topographical heatmapping of the relevant protein log_2_ fold‐changes onto the cryogenic electron microscopy (cryo‐EM) structure of the human mitoribosome illustrated a subunit specific destabilisation of the mtSSU (Figure 2B), resulting in a differential expression pattern consistent with previously reported individuals harbouring biallelic pathogenic variants in other nuclear genes associated with the mtSSU [14, 15, 16]. Relative Complex Abundance (RCA) analysis was carried out to quantify the relative abundance of OXPHOS and mitoribosome complexes (Figure 2C). RCA analysis quantified mtSSU levels to be significantly reduced to 33% of control levels, while the mtLSU remained unchanged. Significant reductions in the abundance of OXPHOS Complexes I and IV were also observed, a major and minor deficiency respectively when applying the Hock et al. [17] criteria, consistent with dysfunctional mitoribosome activity adversely impacting mitochondrial translation.

## 3 Discussion

Mitochondria play an essential role in embryogenesis and organogenesis, as demonstrated by embryonic lethality in several mitochondrial disease gene knockout animal models [18], including a *Mrps22* mouse model [11]. This reflects both the high energy demands of early fetal development and the broader role of mitochondria in cellular homeostasis and cell fate decisions [19]. While mitochondrial disease can manifest at any age, in utero presentation is less commonly recognised. Reported antenatal features include fetal growth restriction, cardiomyopathy, hydrops, oligohydramnios, microcephaly and a range of brain anomalies (ventriculomegaly, corpus callosum agenesis, cerebellar hypoplasia, and cystic changes) [7, 20, 21, 22, 23, 24, 25, 26]. Including the present report, at least 12 cases from seven families with severe MRPS22‐related mitochondrial disease have now been described (Table 1) [5, 6, 7, 8, 9, 10]. Antenatal onset is reported in the majority, with neurological involvement a consistent feature. Corpus callosal abnormalities were present in five families and frequently accompanied by additional brain anomalies such as periventricular cystic changes, ventriculomegaly and delayed gyration. Other common findings included hydrops fetalis (3/7 families), cardiac hypertrophy (5/7 families), microcephaly (3/7 families), and in liveborn cases, profound hyperlactatemia (4/4 families) and hyperammonaemia (2/4 families). Renal involvement, pulmonary hypoplasia and dysmorphic features were variably reported.

**TABLE 1:** Phenotype and genotype of reported early‐onset MRPS22‐related mitochondrial disease cases.

References	Saada et al. [5]			Smits et al. [10]	Baertling et al. [9]	Kilic et al. [8]	Stals et al. [6]	Boutaud et al. [7]			This study
Cases	1a^a^	1b^a^	1c^a^	2	3	4	5 (2 or more pregnancies)^b^	6a^c^	6b^c^	6c^c^	7
Onset of features	Antenatal	Antenatal	Antenatal	Antenatal	2 days	1 day	Antenatal	Antenatal	Antenatal	Antenatal	Antenatal
Age of death	2 days	22 days	NS	Alive at 5.5 years	3 days	Alive at 4 years	NS	TOP 35/40	TOP 23/40	TOP 27/40	TOP 21 + 5/40
Sex	Female	Female	Female	Male	Male	Male	NS	Female	Female	Male	Female
Hydrops	Yes	Yes	Yes	No	No	NS	Yes	NS	NS	NS	Yes
Microcephaly	NS	NS	NS	Yes	NS	NS	NS	Yes	Yes	Yes	Yes
CC abnormality	NS	NS	NS	CC hypoplasia	CC agenesis	NS	CC agenesis	CC dysplasia	CC dysplasia	CC dysplasia	CC agenesis
Other brain findings	NS	NS	NS	Dilatation of the third ventricle, subdural haematoma, leukoencephalopathy and delayed myelination	Multiple periventricular cysts and small hyperechoic foci	Bilateral, symmetrical T2 hyperintensities in the brainstem and medial thalamus with diffusion restriction, progressive cerebral volume loss	Ventriculomegaly	Germinolytic cysts, projection fibre anomalies, gliosis, basal ganglia and pontine hypoplasia	Enlarged germinal zones and germinolytic cysts, projection fibre anomalies, basal ganglia and pontine hypoplasia	Delayed gyration, enlarged germinal zones and germinolytic cysts, projection fibre anomalies, basal ganglia and pontine hypoplasia	Periventricular cysts, widespread intracranial haemorrhage, small cerebellum, prominent cisterna magna
Cardiac	HCM	HCM	HCM	Biventricular HCM, PHN, WPW	ASD, VSD, PHN, coronary artery fistula	No	HCM	Dysplastic valves, right hypertrophy	Right hypertrophy, inter‐auricular canal	NS	Thickened myocardium
Lung	NS	NS	NS	NS	NS	Recurrent aspiration pneumonia, tracheostomy with mechanical ventilation	Pulmonary hypoplasia	NS	NS	NS	Pulmonary hypoplasia
Kidneys	Tubulopathy	Tubulopathy	Tubulopathy	NS	NS	No	NS	NS	NS	NS	Mild pelvicalyceal dilatation
Dysmorphism	NS	NS	NS	Synophrys, low, posteriorly rotated ears, retrognathia, redundant neck skin hypospadias	NS	High arched palate, hypertelorism, broad eyebrow, round face, prominent ears	Low‐set ears, wide anterior fontanel	Coarse facies	Coarse facies	Coarse facies, synophrys	NA
Hypotonia	Yes	Yes	Yes	Yes	Yes	Yes	NS	NA	NA	NA	NA
Developmental delay	NA	NA	NA	Yes—severe	NA	Yes—severe	NS	NA	NA	NA	NA
Tetraspasticity	NA	NA	NA	Yes	NA	Yes	NS	NA	NA	NA	NA
Other features	No	No	No	Dysphagia, FTT seizures	No	Dysphagia	NS	Bilateral toes ectrodactyly	Hypertrophy external genital organs, septate uterus	Hypospadias, camptodactyly, broad hallux	
Biochemical	Lactic acidosis, hyperammonaemia	Lactic acidosis, hyperammonaemia	NS	Lactic acidosis, elevated urinary Krebs cycle intermediates	Lactic acidosis, hyperammonaemia	Lactic acidosis, elevated urinary Krebs cycle intermediates.	NA	NA	NA	NA	NA
RCE	CI, CIII, CIV and V reduced to 80%–30% of controls (mus)	Complexes slightly reduced in lymphocytes (47%–62%)	NS	CI, III and IV reduced to 45.59% and 36% of lowest control (fb)	Reduced activity of CI, III, IV in fb		NA	NA	NA	NA	NA
Genotype	c.509G > A;p.(Arg170His)/c.509G > A;p.(Arg170His)	c.509G > A;p.(Arg170His)/c.509G > A;p.(Arg170His)	c.509G > A;p.(Arg170His)/c.509G > A;p.(Arg170His)	c.644 T > C;p.(Leu215Pro)/c.644 T > C;p.(Leu215Pro)	c.1032_1035dup;p.(Leu346Asnfs*21)/c.1032_1035dup,p.(Leu346Asnfs*21)	47,XY,+ 21[4]/46,XY[46], c.339 + 5G> A/c.339 + 5G > A	c.509G > A;p.(Arg170His)/c.878 + 1G > T	c.648 + 1G > T/c.758 T > C;p.(Ile253Thr)	c.648 + 1G > T/c.758 T > C;p.(Ile253Thr)	c.648 + 1G > T/c.758 T > C;p.(Ile253Thr)	c.509G > A;p.(Arg170His)/c.565C > G;p.(Arg189Gly)

Abbreviations: ASD, atrial septal defect; CC, corpus callosum; FTT, failure to thrive; HCM, hypertrophic cardiomyopathy; NS, not specified; NA, not applicable; PHN, pulmonary hypertension; RCE, respiratory chain enzymology; TOP, termination of pregnancy; VSD, ventricular septal defect; WPW, Wolff Parkinson White syndrome.

^a^ Siblings.

^b^ Specific number of affected pregnancies not reported.

^c^ Siblings.

This review underscores a recognisable, though non‐specific, severe antenatal phenotype with most cases resulting in termination or neonatal death. Pregnancy loss is devastating for families, particularly when the underlying cause and recurrence risk remain uncertain [27]. Antenatal mitochondrial disease is challenging to diagnose based on phenotype alone, given the non‐specific findings and phenotypic overlap with non‐mitochondrial diseases [28]. Genomic autopsy is increasingly valuable in this context, utilising a broad analysis approach [29]. One cohort reported a molecular diagnosis from genomic autopsy in 21% of families with fetal or neonatal death when standard autopsy was inconclusive, with a further 5% requiring additional functional studies to resolve VUS [30]. Next‐generation sequencing has expanded our capacity to detect variants, but interpretation remains challenging, especially in the antenatal setting where the evolution of the clinical phenotype cannot be assessed or tissue availability may be limited. Untargeted quantitative proteomics has emerged as a powerful approach to functionally validate variants at scale, with demonstrated utility for mitochondrial disease, although it is not yet available clinically [17]. Many mitochondrial disease variants affect the assembly or stability of multimeric complexes, including the mitoribosome and OXPHOS complexes. Variants encoded within structural subunits or assembly factors can lead to protein complex destabilisation, readily detectable by proteomic analysis. Quantification using Relative Complex Abundance (RCA) has been shown to have greater sensitivity than clinical respiratory chain enzymology for detecting mitochondrial defects [17, 31, 32, 33]. Functional proteomics has broader applicability to other rare monogenic disorders given the ability to quantify proteins encoded by over half of the Mendeliome disease genes in a single test [17, 34] and return results in a clinically actionable timeframe [17]. Integration of proteomics into clinical diagnostic laboratories could represent a transformative and cost effective [35] advancement in the diagnostic evolution of both mitochondrial and other rare genetic diseases. However, implementing proteomics at scale would require standardised protocols to minimise inter‐laboratory discrepancies, larger control cohorts to better define ranges of normal versus affected protein and complex abundances, and dedicated funding to support the capital and operational costs for instrumentation as well as specialised laboratory and computational infrastructure [36].

In this case, the fetus had compound heterozygous variants in *MRPS22*; a previously reported pathogenic missense variant (c.509G>A;p.Arg170His) and a missense VUS (c.565C>G; p.Arg189Gly). No clear genotype–phenotype correlation is evident in the limited number of cases reported, with missense, splice, and frameshift variants all associated with severe disease. Pathogenic variants in *MRPS22* destabilise the mitoribosome, leading to mitochondrial protein translation defects and combined OXPHOS deficiencies (Table 1) [5, 7, 9, 10]. Our proteomics data supported this mechanism, showing a significant and isolated destabilisation of the mtSSU, resulting in a specific pattern consistent with other mitoribosome‐related diseases [14, 15, 16, 33, 37] and consequent reduction in multiple OXPHOS complex abundances. The functional evidence in this case was pivotal, which combined with a turnaround time of 1 week from sample receipt to reporting, enabled reclassification of the VUS, confirming the molecular diagnosis. While this turnaround time was possible due to the availability of an established fibroblast cell line, other sample types such as peripheral blood mononuclear cells (PBMC) and tissue can be rapidly obtained and show similar utility to fibroblasts in detection of OXPHOS deficiencies using proteomics [17]. Moreover, respiratory chain enzymology would only provide functional information regarding respiratory chain activities that would support but not directly confirm a mitoribosome assembly defect, thus additional targeted testing required for sufficient evidence of variant pathogenicity would not be achievable in the same timeframe.

There is currently no targeted therapy for MRPS22‐related mitochondrial disease. Although pre‐clinical studies in patient fibroblasts demonstrated modest biochemical improvements with 5‐aminoimidazole‐4‐carboxamide ribotide, ascorbic acid, and *N*‐acetylcysteine, clinical benefit has not been demonstrated [8, 38]. Given the poor prognosis with embryonic and early infantile lethality, the value of a diagnosis lies in providing accurate genetic counselling and reproductive options for affected families [39]. Following pregnancy loss, time is of the essence to provide answers for families. Usually targeted, low‐throughput functional studies take considerable time to validate variants of uncertain significance, if available at all. In our case, rapid functional validation of the *MRPS22* variants and thus accurate genetic counselling allowed the family to proceed to prenatal testing for a subsequent pregnancy within a year. This outcome illustrates the tangible clinical utility of timely genomics and proteomics in rare disease diagnosis.

## 4 Methods

### 4.1 Genome Sequencing, Analysis and Interpretation

Fetal DNA was extracted from post‐mortem fetal lung tissue. Parental DNA was extracted from whole blood. Trio genome sequencing was performed as previously described [30] at the clinically accredited SA Pathology ACRF Cancer Genomics facility, on the Illumina NovaSeq Sequencing System, following PCR free ISO15189 Medical testing workflows with a mean coverage of 30×. Sequences were aligned to the human reference genome (UCSC GRCh38/hg38) and variants were called using DRAGEN v4.2. Variant annotation was performed using VariantGrid v3 and Illumina Emedgene v35. A phenotype‐driven analysis was performed utilising Human Phenotype Ontology terms and virtual gene lists available on PanelApp Australia [40](https://panelapp‐aus.org/). Variant classification was based on American College of Medical Genetics and Genomics reporting guidelines [41].

### 4.2 Quantitative Proteomics

Whole cell fibroblast pellets from the proband and five paediatric controls with no known or suspected mitochondrial OXPHOS defect were solubilised in lysis buffer containing 5% (w/v) SDS and 50 mM tetraethylammonium bromide (TEAB) pH 8.5, and quantified using the Pierce BCA Protein Assay Kit (Thermo Fisher Scientific). A total of 25 μg of protein was aliquoted in triplicate for the patient and a single replicate for each control, followed by processing by S‐Trap micro spin columns (Protifi) as per the manufacturer's instructions. Proteins were digested into peptides with trypsin (Thermo Fisher Scientific) at a 1:25 trypsin to protein ratio and dried down using a CentriVap Benchtop Vacuum Concentrator (Labconco). Peptides were reconstituted in 2% (vv) acetonitrile (ACN) and 0.1% (v/v) trifluoroacetic acid (TFA) for analysis by liquid chromatography (LC)‐tandem mass spectrometry (MS/MS) on an Orbitrap Astral Mass Spectrometer (Thermo Fisher Scientific) coupled to a Vanquish Neo UHPLC system (Thermo Fisher Scientific), operating in a data independent acquisition (DIA) mode over a 30 min gradient as described in [32].

Raw data were processed using Spectronaut (v.19.0.240606.62635, Biognosis) against a UniProt human database containing 42 360 canonical and isoform protein entries using a library free directDIA+ (deep) workflow. Default BGS Factory Settings were used with the following modification: ‘Precursor PEP Cutoff’, ‘Protein Qvalue Cutoff (Run)’, and ‘Protein PEP Cutoff’ were all set to 0.01, ‘Exclude Single Sit Proteins’ was selected, and both ‘Major Group Top N’ and ‘Minor Group Top N’ were deselected. Raw MS2 Quantities were imported into Perseus (version 1.6.15.0) [42] and filtered for at least 2 valid values across both patient and control replicates. MS2 quantities were log_2_ transformed and a two‐sided *t*‐test was performed and visualised using the scatter‐plot function, with significance set to *p‐*value = 0.05 (−log_10_ = 1.301) and fold‐change = +1.5 (log_2_ = 0.585) (Supporting Information S1). Small and large subunit proteins of the mitochondrial ribosome (mitoribosome) were annotated using MitoCarta3.0 [43], with the exception of MRPS36 which was annotated as a mtSSU protein but is a known mitoribosome misnomer. Relative Complex Abundance (RCA) was calculated and plotted for OXPHOS Complexes I–V and the mitoribosome using R (version 4.3.0) and Rstudio (2024.04.2 + 764) using an in‐house script [17]. Topographical heatmap was generated by plotting the log_2_ fold‐change abundance of each mitoribosome protein generated from the *t‐*test above on the structure of the human mitoribosome (PDB: 3J9M) [2] using an in‐house script [44].

## Author Contributions

Patient care: A.W., J.K., J.L.; data collection: J.L., M.B.; data analyses/interpretation: L.D.J, L.N.S., D.H.H., D.A.S., A.G.C., D.R.T., J.C.; manuscript drafting and revising: L.N.S., M.B. All authors read and approved the final manuscript.

## Funding

This research was supported by Australian Medical Research Future Fund Genomics Health Futures Mission grants (2007959 to D.R.T., 2016030 to D.A.S.), an Australian National Health and Medical Research Council (NHMRC) Investigator Fellowship (2009732 to D.A.S.) and a Principal Research Fellowship (1155244 to D.R.T.). We thank the Mito Foundation for a research grant (G067 to DRT) and the provision of instrumentation through research equipment grants (G189) to D.A.S. and D.H.H., and a PhD Top‐Up Scholarship to L.N.S. (S021) and M.B. (S037). The research conducted at the Murdoch Children's Research Institute (MCRI) was supported by the Victorian Government's Operational Infrastructure Support Program. The Chair in Genomic Medicine awarded to JC is generously supported by The Royal Children's Hospital Foundation.

## Ethics Statement

This family provided informed written consent and was enrolled in the national research program MitoMDT. This study was conducted in accordance with the revised Declaration of Helsinki and following the Australian National Health and Medical Research Council statement of ethical conduct in research involving humans. This study has Human Research Ethics Committee approval (HREC/82160/RCHM‐2022).

## Consent

This family provided informed written consent for publication.

## Conflicts of Interest

The authors declare no conflicts of interest.

## Supporting information


**Supporting Information: S1** Proteomic dataset of two‐sided *t*‐test results comparing proband fibroblasts against controls (*n* = 5).

## Data Availability

The datasets used and/or analysed during the current study are available from the corresponding author on reasonable request.

## Appendix A MitoMDT Diagnostic Network for Genomics and Omics Consortium

David R. Thorburn, Aleksandra Filipovska, Michael T. Ryan, David A. Stroud, Diana Stojanovski, David Coman, Sean Murray, Ryan L. Davis, John Christodoulou, Suzanne C. E. H. Sallevelt, Roula Ghaoui, Cas Simons, Stefan J. Siira, Shanti Balasubramaniam, Alison G. Compton, Daniel G. MacArthur, Nicole J. Lake, Drago Bratkovic, Joy Lee, Maina Kava, Amanda Samarasinghe, Yoni Elbaum, Catherine Atthow, Pauline McGrath, Tegan Stait, Rocio Rius, Liana N. Semcesen, Megan Ball, Daniella Hock, Luke E. Formosa, Ellenore M. Martin, Madeleine Harris.
